# Study on the application of progressive training method combined with imagery training method in laparoscopic suturing skills training for resident physicians

**DOI:** 10.1186/s12909-025-06928-y

**Published:** 2025-03-12

**Authors:** Wenxue Lin, Jian Yu, Xiaoping Peng, Jianfu Xia, Bingchen Huang, Rizeng Li

**Affiliations:** 1 Department of oncology Surgery, The Dingli Clinical College of Wenzhou Medical University (Wenzhou Central Hospital), Wenzhou, China; 2 Department of General Surgery, The Dingli Clinical College of Wenzhou Medical University (Wenzhou Central Hospital), Wenzhou, China; 3 Department of Surgery, The Dingli Clinical Institute of Wenzhou Medical University, Wenzhou, China; 4 Clinical Skill Center, The Dingli Clinical College of Wenzhou Medical University (Wenzhou Central Hospital), Wenzhou, China

**Keywords:** Laparoscopic suturing skills, Intracorporeal suturing and knot-tying, Progressive training method, Imagery training method, Resident physicians, Laparoscopic simulation training

## Abstract

**Objective:**

This study aims to explore an efficient teaching method to improve laparoscopic suturing skills in resident physicians by combining the progressive training method with the imagery training method.

**Methods:**

This study used a randomized controlled trial methodology. The experimental group received training utilizing a combination of the progressive training method and the imagery training method In contrast, the control group followed the traditional teaching method of practicing continuous complete actions. Both groups were trained in intracorporeal suturing and knot-tying under laparoscopy. The training effects of the two groups were compared and analyzed before and after the training, including LS-CAT scores and suturing time.

**Results:**

In the second test, the experimental group had much higher LS-CAT scores than the control group, as well as a considerably lower number of operation errors. In the experimental group, 88.9% of the trainees were proficient, compared to only 28.6% in the control group. There was no significant difference in suturing time between the two groups. The results of the third test indicated that, although there were no significant differences in LS-CAT total scores or suturing time between the two groups, the experimental group demonstrated a significantly lower number of operational errors compared to the control group. Additionally, the LS-CAT scores for tissue handling in the experimental group were significantly better than those in the control group.

**Conclusion:**

The combination of the progressive training method and the imagery training method significantly improved resident physicians’ laparoscopic suturing skills. This method greatly enhanced the efficiency and quality of learning laparoscopic suturing and knot-tying skills among surgical and gynecological resident doctors.

## Introduction

A well-known and considerable positive correlation exists between laparoscopic simulation training and intraoperative performance [1]. Laparoscopic suturing skills are one of the core competencies required of surgeons and represent a challenging element of laparoscopic simulation training. Traditional teaching methods for laparoscopic suturing skills usually involve instructing and practicing continuous, complete actions. This approach results in high learning difficulty, causing trainees to experience learning stagnation at critical points, leading to low training efficiency and limited learning outcomes. Beginners often confront a steep learning curve to overcome the bottleneck in laparoscopic suturing skills.

Additionally, the extended learning curve for laparoscopic surgeons can prolong patient surgery times, increase the risk of surgical complications [2], and place a greater strain on public finances [3]. The progressive training method-based approach to teaching laparoscopic suturing skills involves breaking surgical skills down into smaller steps, allowing learners to master each skill step by step gradually. This method enables learners to continually drill individual challenging points, boosting overall proficiency and accuracy.

In China, Jin Yang and colleagues applied the progressive training method to simulated training for laparoscopic pancreatojejunostomy and achieved significant results [4]. In Japan, Mizota T and Kurashima Y successfully applied this method in basic laparoscopic skills training [5].

The imagery training method involves mental training in which students repeatedly simulate the skill operation process in their minds, thereby deepening their memory and understanding of the skills. This method also encourages learners to quickly reflect on and adjust their operations, continuously improving their skill level. The imagery training method has been widely employed in sports training, such as golf putting training, where two action states are simulated to achieve cognitive adaptation and skill progress [6]; Mental practice has been shown to improve performance in dart throwing [7]. Furthermore, the imagery training method has been widely applied in the rehabilitation of neurological disorders such as stroke [8–11]. Could these experiences be extended to surgery? Currently, studies specifically evaluating the effectiveness of imagery training in surgical training are relatively scarce [12], warranting further research and validation.

This study aims to explore a more efficient teaching method by combining progressive training with imagery training to enhance laparoscopic suturing skills in resident physicians.

## Materials and methods

### Ethical considerations

This randomized controlled trial was approved by Wenzhou Central Hospital’s Medical Ethics Committee(no.202402270046000397799).

All participants were given detailed explanations about the study and submitted written informed consent.

### Equipment and materials

The study utilized laparoscopic simulation training box with internal high-definition camera ( GD/W-200 laparoscopic surgery simulation training systems from Shanghai Honglian Medical Technology Group ). Suture modules with silicone simulation skin and suture lines with needles (“3 − 0”, 15 cm) were used.

### Study design

This study adopted a randomized controlled design. The study subjects were 16 resident doctors from the 2202 cohort of the standardized training program for surgical and obstetrics-gynecology bases at Wenzhou Central Hospital, along with one newly employed surgical resident. Before the implementation of the study, all enrolled trainees had not received any simulation training in laparoscopic suturing skills. The researchers randomly assigned the participants to two groups: the experimental group, comprising 9 participants who used the progressive training method combined with the imagery training method, and the control group, comprising 8 participants who used the comprehensive training method. One trainee in the control group adjusted their training plan due to conflicts between work and study schedules, and the final analysis did not include their data.

### Experimental protocol

The study’s training skill was the intracorporeal suturing and knot-tying skill based on the Fundamentals of Laparoscopic Surgery (FLS) [13]. During the study, the researchers held training sessions once a week for over 3 weeks, each lasting 100 min, totaling 300 min. Researchers tested the trainees’ intracorporeal suturing and knot-tying skills before, during (at 150 min into the training), and after (300 min after) the training. Each test was recorded using the simulation box’s built-in video recording function, with a designated person in charge of collecting the videos.

#### 4.1

The experimental group utilized a training method combining the progressive and imagery training methods.

#### 4.1.1

In the progressive training method, students will practice each decomposed action step by step during the instructor-led teaching and subsequent training sessions, focusing on individual challenges. The complete suturing process is broken down into six steps: needle positioning, needle handling, needle entry and exit, knot tying 1 (looping the thread), knot tying 2 (tightening the knot), and cutting the thread. Trainees will practice each step progressively until they are proficient. After practicing all decomposed actions (during the first and second weeks), the trainees will enter a phase of continuous laparoscopic suturing technique training (the third week).

#### 4.1.2

The training schedule for the imagery training method is as follows: Before practicing each decomposed action and before the continuous laparoscopic suturing technique training, trainees will undergo 2 min of relaxation and imagery training. In the 100-minute sessions during the first and second weeks, trainees will perform 2 min of imagery training before each of the six steps, totaling 12 min of imagery training per session. The third week focuses on continuous laparoscopic suturing technique training, with 2 min of imagery training before each session, where trainees typically complete 8.7 continuous suturing sessions, averaging 17.4 min of imagery training. The content of the imagery training includes total body relaxation, light eye closure, and visualization of correctly performing each step of laparoscopic suturing inside the body, guided by the instructor’s cues. Therefore, our imagery training protocol lasts 12 to 17.4 min per session, conducted three times in total. Besides formal course training, we neither specifically encourage nor restrict trainees from practicing imagery training at other times.

#### 4.2

The control group used traditional teaching methods, practicing continuous and complete intracorporeal suturing and knot-tying techniques during the instructor’s lectures and subsequent training sessions.

### Outcome assessment

Two experts evaluated and rated the videos using the Laparoscopic Suturing Competency Assessment Tool (LS-CAT) [14]. Throughout the grading process, the experts were kept unware of the participants’ identities. The LS-CAT grading system featured four task areas: needle handling and adjustment, needle insertion and withdrawal through tissue margins, correctly tying the first surgical knot, and knot-tying. For each task area, the LS-CAT evaluates performance based on instrument handling, tissue manipulation, and the number of operational errors, using a scoring scale from 1 to 4. A perfect score of 8 indicates flawless performance. Additionally, there are 16 error scoring items. Lower LS-CAT scores indicate higher proficiency and competency of the participants. Fewer operational errors also suggest higher proficiency and competency, as well as better suturing quality.

Additionally, we also recorded how long it took to complete the intracorporeal suturing and knot-tying. Furthermore, we used the Likert scale to collect data on trainees’ confidence levels, involvement in imagery training, and self-perceived ability to perform imagery actions before and after training. Data on trainees’ learning experiences with the progressive training method, skill improvement, the effectiveness of the imagery training method, and overall teaching satisfaction were also collected.Imagery training involvement encompasses five aspects: being able to vividly imagine the suturing process in laparoscopic surgery; feeling the sensation of holding surgical instruments during imagery training; mentally reproducing the entire sequence of steps in laparoscopic suturing; being able to fully concentrate during imagery training without distraction; and believing that imagery training is a useful method for improving laparoscopic surgical skills.

### Statistical analysis

All data were analyzed using SPSS 24.0. The researchers assessed baseline characteristics of the participants with Fisher’s exact test and the Mann-Whitney U test, and they used the Kruskal-Wallis H test to compare LS-CAT scores and error counts across the three tests. The Mann-Whitney U test was used to compare changes in medical confidence, action imagery, and participation in imagery training before and after training, as well as differences in LS-CAT scores and operation error counts between the two groups. Medians (interquartile range, IQR) are used to express quantitative values. P-values < 0.05 were considered statistically significant.

## Results

1. 17 surgical and obstetrics-gynecology resident doctors participated in this study. One trainee adjusted their training plan due to conflicts between work and study schedules, and their data were not included in the study. Researchers randomly assigned sixteen participants to the experimental group and the control group. There were no significant differences in baseline characteristics between the two groups. See Table 1.
2. All trainees’s LS-CAT scores and operation error counts were compared in the first, second, and third tests. The results showed significant improvements in all trainee’s laparoscopic LS-CAT scores and operation error counts in the second and third tests. See Table 2.

**Table 1:** Baseline characteristics of participants

Characteristic	Experimental Group (*n* = 9)	Control Group (*n* = 7)	*P* Value
Number of Participants	9	7	
Male (n)	5	3	1
Female (n)	4	4	
Bachelor’s Degree (n)	6	6	0.585
Master’s Degree (n)	3	1	
Age (years)	28 (26.5, 29)	27 (26, 27)	0.142
BMI	20 (18.6, 22.7)	20.9 (18.6, 24.5)	0.47
First Test LS-CAT Total Score (points)	24 (22, 25.5)	24 (22, 25)	0.837
First Test Suturing Time(seconds)	505(405.5, 587.5)	418(340, 515)	0.4
Lapar0scopic Surgery Experience(times)	17(9, 31)	8(6, 24)	0.142

**Table 2:** LS-CAT scores and operation error counts of participants

Group	Category	First Test	Second Test	Third Test	H	*P*
Experimental Group	Total Score (points)	24.0 (22, 25.5)	13.0 (12, 14)	11.0 (9, 12)	21.2	< 0.001
	Instrument Handling	12.0 (11, 13)	7.0 (6, 7)	6.0 (5, 6)	20.5	< 0.001
	Tissue Handling	12.0 (11, 12.5)	7.0 (6, 7)	5.0 (4, 6)	21.3	< 0.001
	Error Count (times)	5.0 (4, 5.5)	1.0 (1, 3)	1.0 (1, 1.5)	19.3	< 0.001
	Suturing Time(seconds)	505(405.5, 587.5)	174(153.5, 198)	140(113, 157.5)	19.4	< 0.001
Control Group	Total Score (points)	24.0 (22, 25)	18.0 (16, 19)	11.0 (10, 15)	16.3	< 0.001
	Instrument Handling	12.0 (12, 13)	9.0 (8, 10)	6.0 (5, 7)	16.9	< 0.001
	Tissue Handling	12.0 (10, 12)	9.0 (8, 10)	6 (5, 8)	14.9	0.001
	Error Count (times)	4.0 (4, 5)	4.0 (3, 4)	3.0 (2, 3)	11.4	0.003
	Suturing Time(seconds)	418(340, 515)	182(155, 222)	104(91, 151)	16.5	< 0.001

H: The Kruskal-Wallis H statistic, which reflects the degree of differences between groups

3. In the second test, the experimental group’s LS-CAT scores and operation error counts were significantly lower than those of the control group. In the third test, the experimental group’s LS-CAT scores and operation error counts in tissue handling were also significantly lower than those of the control group. There were no significant differences in the LS-CAT total scores and suturing times between the two groups. See Tables 3 and 4.

**Table 3:** Comparison of LS-CAT scores in the second test between experimental and control groups

Category	Experimental Groups	Control Groups	*P*
LS-CAT Total Score (points)	13.0(12, 14)	18.0(16, 19)	0.005
LS-CAT Instrument Handling Score	7.0(6, 7)	9.0(8, 10)	0.008
LS-CAT Tissue Handling Score	7.0(6, 7)	9.0(8, 10)	0.003
Operation Error Count (times)	1.0(1, 3)	4.0(3, 4)	0.001
Suturing Time(seconds)	174(153.5, 198)	182(155, 222)	0.536

Numerical values are represented by the median (inter quartile range, IQR), and the statistics are based on the Mann-Whitney U test

**Table 4:** Comparison of LS-CAT scores in the third test between experimental and control groups

Category	Experimental Groups	Control Groups	*P*
LS-CAT Total Score (points)	11.0(9, 12)	11.0(10, 15)	0.21
LS-CAT Instrument Handling Score	6.0(5, 6)	6.0(5, 7)	0.536
LS-CAT Tissue Handling Score	5.0(4, 6)	6(5, 8)	0.042
Operation Error Count (times)	1.0(1, 1.5)	3.0(2, 3)	0.003
Suturing Time(seconds)	140(113, 157.5)	104(91, 151)	0.174

Numerical values are represented by the median (interquartile range, IQR), and the statistics are based on the Mann-Whitney U test

4. The surgical confidence scores of trainees in the experimental group significantly improved after training, and the imagery training participation scores of the experimental group also showed a significant increase. See Table 5.

**Table 5:** Comparison of confidence and imagery scores before and after training

Group	Category	Before Training	After Training	z	*P*
Experimental Group	Surgical Confidence Score (points)	26.0 (20.5, 34.5)	36.0 (30.5, 40)	2.4	0.014
	Action Imagery (points)	24.0 (19.5, 25)	28.0 (22.5, 30)	1.6	1.36
	Imagery Training Participation (points)	20.0 (18, 23.5)	24.0 (22, 25)	2.2	0.031
Control Group	Surgical Confidence Score (points)	27 (23, 29)	29 (26, 31)	1.3	0.209

## Discussion

Laparoscopic surgical skills training has long been a global medical education and surgical research focus. Studies have shown that standardized laparoscopic surgical simulation training can reduce surgical complications and the risk of conversion to open surgery [15, 16]. Among these skills, intracorporeal suturing and knot-tying under laparoscopy are recognized as one of the most challenging aspects of laparoscopic surgical simulation training. Traditional teaching methods involve instructing and practicing continuous, complete actions. However, resident physicians often experience learning stagnation at critical points due to high learning difficulty, leading to low training efficiency and prolonged learning curves.

Additionally, the current equipment and funding for laparoscopic training are limited [17]. In light of increasing hospital operating costs, finding ways to help trainees focus on training steps suitable for their skill levels, thereby smoothly overcoming learning bottlenecks, improving the learning outcomes and efficiency of laparoscopic suturing skills, and saving training costs, has become an urgent issue.

This study compared the differences in learning outcomes and efficiency of laparoscopic suturing skills between this new teaching method, which combines the progressive and imagery training with traditional teaching methods.

In the second test, the experimental group significantly outperformed the control group in LS-CAT scores and had noticeably fewer operational errors, though no significant differences in suturing time were observed between the groups.

The results of the third test showed that although there were no significant differences in LS-CAT total scores and suturing times between the groups, the experimental group continued to have significantly fewer operational errors and better scores in tissue handling on the LS-CAT. This suggests that the new teaching method not only improved the precision and quality of laparoscopic suturing but also effectively reduced operational errors.

These improvements demonstrate significant advantages, particularly in enhancing the accuracy and quality of laparoscopic suturing skills. In surgery, the quality of suturing is crucial; poor or inconsistent suturing quality can undermine the success of an operation, regardless of the speed of suturing.

Thus, the new teaching method played a key role in training students in high-quality suturing skills. The advantages of the experimental group were clear in the second test after 150 min of training, and while the performance of the two groups began to converge in the third test after 300 min of training, the experimental group still maintained its advantage in the number of operational errors and tissue handling scores. This indicates that the new teaching method not only effectively improves the level of laparoscopic suturing skills but also has distinct advantages in teaching efficiency.

The progressive training method breaks down the complex laparoscopic suturing operations into simple, manageable segments, allowing trainees to advance progressively from simple to complex tasks and to repeatedly practice specific challenging points. This approach helps trainees adapt to high-difficulty operations, gradually building complete skills, and significantly improves learning efficiency. The progressive training method has been widely applied in related surgical fields such as laparoscopy and colonoscopy [18–20]. In Japan, Tomoko Mizota and colleagues found that a step-by-step training method with remote guidance for laparoscopic suturing skills training could more effectively utilize the time of both trainees and trainers [21]. Further studies by Chen HA and Huang SW confirmed that medical students and surgical trainees who used the progressive training method improved their laparoscopic suturing skills [22] significantly. These findings are consistent with the results of this study.

Imagery training, also known as motor imagery training, has been proven to effectively improve motor performance and enhance participants’ skill levels in various fields such as sports psychology, neurological rehabilitation, and surgical cholecystectomy [8, 23–25]. However, its application in surgical education still suffers from a lack of data. Currently, psychological motor skills such as visuospatial abilities and perceptual capabilities have been proven to be closely related to surgical skill levels [26, 27]. This study introduces motor imagery training, using mental practice to repeatedly simulate skill operations in the mind, thereby deepening the memory and understanding of skills. The experimental group showed significant improvement in the involvement in imagery training, and the method was actively accepted and participated in by the students, indicating significant advantages in helping students form clearer surgical visualizations, enhance concentration, and improve training outcomes.

Although the duration of each imagery training session in this study was 12 to 17.4 min, with a total of three sessions conducted at a low frequency, the active participation of the experimental group in motor imagery training may be related to their actual improvement in operational skills, which is worth further verification in subsequent studies. Mary S. L. Goble and others in their review have noted that based on the successful experiences in the field of neurological rehabilitation, subjects with strong intrinsic motivation can significantly improve learning outcomes [12]. In surgery, mastering laparoscopic suturing techniques is a basic skill and core competency for surgeons. Trainees in this training have clear professional goals aimed at enhancing medical skills, and this goal-oriented motivation can effectively stimulate the intrinsic learning drive of surgical trainees, potentially enhancing the effects of motor imagery training, which is worth further exploration in future research. Additionally, trainees in the experimental group showed significantly higher surgical confidence after training than before, indicating that the new training method not only improves surgical skills but also enhances their confidence.

This study has several limitations. First, although progressive training and imagery training have been applied in their respective fields, studies on their combined application are still rare. Therefore, the initial intention of this study was to fill this research gap. However, due to the inability to independently quantify the effects of each method, future studies should further explore the independent effects and interactions of each method. Secondly, the small sample size of this study may lead to an amplification of Type II errors, thereby reducing the power of the statistical results. Therefore, future research should increase the sample size and consider extending the study to a clinical setting to further validate our hypotheses and conclusions.

## Conclusion

In summary, the study demonstrates that using an innovative combination of progressive training and imagery training significantly enhances laparoscopic suturing skills among resident physicians. This approach notably improves the efficiency and quality of suturing during laparoscopic knot tying for surgical and gynecological residents, reducing the resources required for training. High-quality laparoscopic suturing skills help decrease operation times and complications, thereby significantly increasing the safety and success rates of surgeries, which directly benefits patients. Additionally, this research provides new ideas and methods for the training of resident physicians in laparoscopic skills, contributing to the reform and innovation of medical education.

## Data Availability

The data and materials during the present research are available from the corresponding author on reasonable request.

## Abbreviations

FLS: Fundamentals of Laparoscopic Surgery
LS-CAT: Laparoscopic Suturing Competency Assessment Tool
IQR: Interquartile range
